# Forecast of Dengue Incidence Using Temperature and Rainfall

**DOI:** 10.1371/journal.pntd.0001908

**Published:** 2012-11-29

**Authors:** Yien Ling Hii, Huaiping Zhu, Nawi Ng, Lee Ching Ng, Joacim Rocklöv

**Affiliations:** 1 Umeå Centre for Global Health Research, Epidemiology and Global Health, Department of Public Health and Clinical Medicine, Umeå University, Umeå, Sweden; 2 Laboratory of Mathematical Parallel Systems, Department of Mathematics & Statistics, York University, Toronto, Ontario, Canada; 3 Environmental Health Institute, National Environment Agency, Singapore, Singapore; DVBNTD/CWRU/Emory University, Kenya

## Abstract

**Introduction:**

An accurate early warning system to predict impending epidemics enhances the effectiveness of preventive measures against dengue fever. The aim of this study was to develop and validate a forecasting model that could predict dengue cases and provide timely early warning in Singapore.

**Methodology and Principal Findings:**

We developed a time series Poisson multivariate regression model using weekly mean temperature and cumulative rainfall over the period 2000–2010. Weather data were modeled using piecewise linear spline functions. We analyzed various lag times between dengue and weather variables to identify the optimal dengue forecasting period. Autoregression, seasonality and trend were considered in the model. We validated the model by forecasting dengue cases for week 1 of 2011 up to week 16 of 2012 using weather data alone. Model selection and validation were based on Akaike's Information Criterion, standardized Root Mean Square Error, and residuals diagnoses. A Receiver Operating Characteristics curve was used to analyze the sensitivity of the forecast of epidemics. The optimal period for dengue forecast was 16 weeks. Our model forecasted correctly with errors of 0.3 and 0.32 of the standard deviation of reported cases during the model training and validation periods, respectively. It was sensitive enough to distinguish between outbreak and non-outbreak to a 96% (CI = 93–98%) in 2004–2010 and 98% (CI = 95%–100%) in 2011. The model predicted the outbreak in 2011 accurately with less than 3% possibility of false alarm.

**Significance:**

We have developed a weather-based dengue forecasting model that allows warning 16 weeks in advance of dengue epidemics with high sensitivity and specificity. We demonstrate that models using temperature and rainfall could be simple, precise, and low cost tools for dengue forecasting which could be used to enhance decision making on the timing, scale of vector control operations, and utilization of limited resources.

## Author Summary

Without effective drugs or a vaccine, vector control remains the only method of controlling dengue fever outbreaks in Singapore. Based on our previous findings on the effects of weather on dengue cases and optimal timing for issuing dengue early warning in Singapore, the purpose of this study was to develop a dengue forecasting model that would provide early warning of a dengue outbreak several months in advance to allow sufficient time for effective control to be implemented. We constructed a statistical model using weekly mean temperature and rainfall. This involved 1) identifying the optimal lag period for forecasting dengue cases; 2) developing the model that described past dengue distribution patterns; 3) performing sensitivity tests to analyze whether the selected model could detect actual outbreaks. Finally, we used the selected model to forecast dengue cases from 2011–2012 week16 using weather data alone. Our model forecasted for a period of 16 weeks with high sensitivity in distinguishing between an outbreak and a non-outbreak. We conclude that weather can be an important factor for providing early warning of dengue epidemics, long term sustainability of forecast precision is challenging considering the complex dynamics of disease transmission.

## Introduction

Dengue fever is a rapidly spreading viral infection that is endemic in more than 100 tropical and subtropical countries in Africa, the Americas, and the Asia Pacific regions. It is caused by any one of the four serotypes of dengue virus, and infection of one serotype of dengue virus does not provide cross immunity against the other three serotypes. Dengue viruses are spread by female *Aedes* mosquitoes through blood-feeding on human hosts. Patients suffering from dengue fever experience sudden onset of fever, rashes, muscle aches, joint pain, and leucopenia. A dengue patient usually recovers within 14 days. Nevertheless, some patients develop severe dengue which is a potentially lethal complication characterized by hemorrhagic manifestations, severe plasma leakage, and severe organ impairment [1]. Globally, about 500,000 severe dengue cases with 12,500 deaths have been reported annually [2].

Singapore has recently experienced an upsurge of dengue incidence with a 5–6 year cyclical epidemic pattern since 1980. The frequency of epidemics has increased in recent years and the nation has experienced four outbreaks over the past eight years (2004–5, 2007, and 2011). During the period 2000–2010, the annual incidence rates of dengue cases per 100,000 populations in Singapore increased from 17 in 2000 to 332 in 2005 before declining to 106 in 2010. Dengue was mainly detected in the eastern region of Singapore before 2004. Subsequently, dengue cases have been reported island-wide with the highest incidence rates in the Central and Southeast dengue zones as demarcated by the National Environment Agency of Singapore (NEA). Over the last decade, the Central and Southeast zones contributed 31% and 25% of total national reported dengue cases, respectively. Reasons for dengue expanding into western region of the island could be complex. Studies have reported that herd immunity among Singapore residents has declined from 47% in the early 90 s to about 29% by 1998; this implies a rise in susceptible populations [3], [4]. A recent seroprevalence study in Singapore showed a ratio of 23 asymptomatic cases to each reported clinical case [5]. These asymptomatic cases can possibly infect the *Aedes* mosquitoes and so form a reservoir of infection. The two main vectors of dengue in Singapore are *Aedes aegypti* and *Aedes albopictus* and studies have shown that they are able to disperse up to the 21^st^ floor of a residential building [6]. All four serotypes of dengue virus (DENV 1–4) have been detected simultaneously in Singapore during the study period, except 2001. DENV 1 was the predominant circulating serotype during the outbreaks in 2004–2005 and DENV 2 was the predominant serotype in 2007 [7]. A study by Lee *et al.* (2012) has suggested that clade replacement in a predominant dengue serotype could also increase dengue incidence in Singapore [8].

Generally, dengue epidemiology is influenced by a complex interplay of factors that include rapid urbanization and increase in population density, capacity of healthcare systems, effectiveness of vector control systems, predominant circulating dengue serotypes, herd immunity, and social behavior of the population. Most dengue endemic countries in Asia Pacific have limited resources and/or lack of preparedness to contain dengue epidemic [9], [10]. Rising international and domestic trade and population movement contribute to the increases in domestic and cross border dengue transmission. As a result, the region is experiencing dengue epidemics with increasing frequency and magnitude. Until a vaccine or drug for dengue is available, vector control operations that eliminate adult mosquitoes and their larvae through breeding-source reduction remain the only effective method to curb dengue transmission. However, vector control can be resource and labor intensive, which poses an economic burden on nations with limited resources.

An early warning system is an essential tool for pre-epidemic preparedness and effectiveness of dengue control. In recent decades, weather variables such as temperature and rainfall have been widely studied for their potential as early warning tools to fend off climate-sensitive infectious diseases such as Malaria, Dengue, and West Nile Virus [11], [12], [13], [14].

Numerous studies have revealed the influence of weather variables on the magnitude of dengue distribution [15], [16], [17], [18], [19], [20] through the effects on life cycle development, biting rates, infective and survival rates of vectors and on the incubation period of dengue virus [21], [22], [23]. As temperature increases, *Aedes* mosquitoes display shorter periods of development in all stages of the life cycle leading to increased population growth; the mosquito feeding rate also increases; and dengue viruses in *Aedes* adult mosquitoes require shorter incubation periods to migrate to salivary glands [21], [22], [24]. Conversely, high temperatures above 35°C or heavy rainfall possibly lower dengue transmission by reducing the survival rate of *Aedes* [21], [23], [25]. Heavy rainfall creates abundant outdoor breeding sources for *Aedes* in the long run, but dry spells in some settings trigger an increase in water storage containers which can serve as breeding habitats.

In recent years, the National Environment Agency of Singapore has been using rising ambient temperature as an indicator of increase in dengue cases. During periods with median ambient temperatures above 27.8°C, the national vector control unit increases surveillance and control operations and the community are urged to increase efforts to reduce mosquito breeding habitats in the relevant residential areas [26]. Nevertheless, a more comprehensive weather-based forecasting tool is required to obtain precise information on the correlation between risk of dengue epidemic and weather conditions favorable for *Aedes* mosquitoes, so that dengue control efforts in the nation can be made more effective in the future.

### Objectives

Previous study by Hii *et al.* (2009) has shown that elevated weekly mean temperature and cumulative rainfall influence the risks of dengue cases in Singapore at lag times up to 20 weeks with higher relative risks of dengue cases at time lag of 3–4 months [15]. Also, a recent study by *Hii et al.* (2012) has suggested that a dengue early warning issues about 3 months in advance could provide sufficient time for an effective mitigation [27]. Based on previous findings, this study aims to develop a simple, precise, and low cost early warning model to enhance dengue surveillance and control in Singapore. Hence, our objectives were first to develop a weather-based dengue forecasting model to project dengue cases or potential outbreak that would allow sufficient time for local authorities to implement preventive measures and second to validate and report the performance of the forecast.

## Materials and Methods

### Study area

Singapore is a highly urbanized island state nation situated at 1°.17′N and 103°.50′E of the equator with a land size of about 700 km^2^. As of 2011, the island accommodates a population of around 5.2 million with about 93% of the population residing in either government or private high rise residential buildings [28]. As a tropical country, Singapore experiences high temperature, rainfall, and humidity year round. Weather in Singapore is influenced by the monsoon rain-belt with highest rainfall between December and early March [29].

### Data collection

Weekly dengue cases from 2000 to 2011 were obtained from the weekly infectious diseases bulletins of Communicable Diseases Division, Ministry of Health (MOH) Singapore [30]. The Infectious Diseases Act in Singapore stipulates mandatory disease notification within 24 hours of diagnosis by all medical clinics and laboratories.

Daily mean temperature and rainfall recorded by the Changi Airport meteorological, southeast of Singapore, for the period of 2000–2011 were extracted from the National Climatic Data Centre, National Oceanic and Atmospheric Administration (NOAA), USA [31]. Weather data were provided to the NOAA by the Meteorological Department of National Environment Agency, Singapore under the regional data collaboration agreement. The daily mean temperature was based on 24 hours average temperature; while daily rainfall was the summation of 24 hours rainfall collected using rain gauges.

### Statistical method

We developed a dengue forecasting model using time series Poisson multivariate regression that allowed over-dispersion of data. Mean weekly predicted cases were estimated through regression on multiple independent variables that include retrospective dengue cases, weekly mean temperature, weekly cumulative rainfall, trend, epidemic cycles and seasonal factors. The forecasting model was developed using three processes: 1) model construction and training using data from 2000–2010; 2) model validation by forecasting cases in 2011–2012; and 3) sensitivity tests on outbreak diagnoses. Our statistical analysis was conducted using R [32] and STATA 11 (2009 StataCorp LP, Texas) based on 95% confidence interval.

### Model construction

We modeled dengue distribution patterns using retrospective data and then extrapolated the patterns several weeks ahead. We developed dengue forecasting models based on assumption that the past dengue distribution patterns will, to a large extent, continue in the future [33]. Bivariate equation (*D_x_*) for each independent variable was first formulated using quasi Poisson regression and subsequently combined to form a multivariate model that takes multiple factors into consideration.
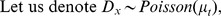
where 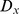 represents weekly average number of predicted dengue cases as a function of independent variable *x*.

### Serial correlation of dengue cases

One characteristic of infectious disease is the influence of past cases on the number of current cases. Therefore, autoregression was included in the model to account for the serial relationship between past and current cases. We derived possible lag time of serial correlation through data analysis using Autocorrelation Function (ACF), Partial Autocorrelation Function (PACF), and prior knowledge on dengue transmission. ACF analysis on dengue data showed gradual decreasing spikes that indicated strong autocorrelation between past and current cases; whereas, PACF cut off after the 4^th^ spike suggesting a lag time of 4 weeks. However, previous studies have shown possible autocorrelation of dengue cases for longer period due to complex reasons that influence the dynamic of dengue transmission [34]. Thus, we examined lag times ranging from 4–12 weeks and selected the optimal lag order using model selection and validation tests.

We denote *D_AR_* as the autoregression of dengue cases k weeks before forecast in week t. The effects of autoregression on dengue cases are computed as:
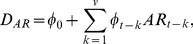
(1)where 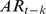 = dengue cases at lag week k,


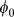
 = the constant number of dengue cases,


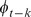
 = parameter of autoregression at lag week k.

### Lag term and meteorological data cycle

We examined the time gap between exposure to weather conditions and subsequent occurrence of dengue cases using cross correlation function and literature reviews. Correlation between temperature and dengue showed sine wave oscillating at about 24-weeks cycle or interval with stronger positive association between lag week 9 and 17. While correlation between rainfall and dengue revealed different length of time cycles with a negative relationship from week 0 to 22. It is possible for dengue transmission to occur several months after favorable weather conditions as mosquito eggs can withstand desiccation for several months with an average egg survival time of 18.3 weeks for *Aedes aegypti* [35]. We identified the optimal lag term and weather time cycle for forecasting by testing lag terms 1–20 weeks with various data cycle periods of weather variables ranging from 12 to 24 weeks. Piecewise regression was used to consider a non-linear relationship between weather and dengue cases. Thus, we partitioned weather data into 4 equally spaced percentiles with knots at 25^th^, 50^th^, and 75^th^ percentiles using spline function.

The impact of weekly weather on dengue cases is estimated as follows:

Let 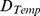
depicts the number of dengue cases as a function of weekly mean temperature:
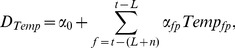
(2)where 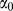
is the baseline number of dengue cases; 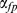 = parameter of mean temperature at lag term *f* in *p* range of mean temperature; f = t - (L+n); *t* = week; *L* = lag term in week; *n* = data cycle period of weekly mean temperature; *p* = temp_11_ to temp_14_ derived from piecewise spline function.

Let 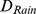 denotes number of dengue cases as a function of weekly cumulative rainfall:
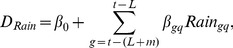
(3)where *β_0_* is the baseline number of dengue cases; 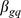 = parameter of rain at lag term *g* in *q* range of weekly cumulative rain; g = t - (L+m); *t* = week; *L* = lag term in week; *m* = data cycle period of weekly cumulative rainfall; q = rain_11_ to rain_14_ derived from piecewise spline function.

### Season, epidemic cycle, and trend

To account for non-climatic factors such as vector control, circulating serotypes of dengue virus, and other factors that influence the number of dengue cases, we performed graphical examination on the trend, cycle, and seasonal distribution patterns of dengue cases over the period 2000–2010. The trend of dengue cases increased with cyclic variation from 2000 to peak at 2005 before declining thereafter. Increases in dengue cases were generally observed in the second half of each year; while major epidemics occurred in 2004–5 and 2007. We included a curvilinear or parabola and sine function to account for trend, epidemic cycle and seasonal influence on dengue cases during the study period, respectively.

Let 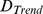 represents dengue cases influenced by trend over the study period:
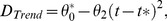
(4)whereas,


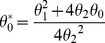
 = constant,


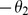
 = parameter measures the trend,

*t* = week,


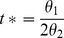
 = point in time where maximal impact of trend is reached.

Let 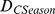 depicts cyclical and seasonal impacts on dengue cases:
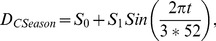
(5)where 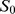 = constant or baseline contribution of cycle and season,


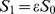
 = parameter that gives rise to cyclical and seasonal effects,

*t* = week.

### Model formulation, selection, and validation

Dengue cases are subject to interactions of multiple complex factors. Thus, we composed a Poisson multivariate regression model by combining equations (1) to (5) to account for influences of multiple factors on dengue cases. We also adjusted our findings for population change by offsetting midyear population (*offset = log (pop)*) during the study period.

Now we summarize our model as follows:
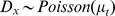
and
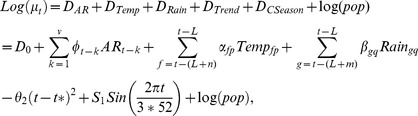
(6)where


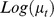
 is the average predicted dengue cases at week *t*,


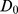
 is the constant derived from multivariate model, and


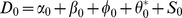
 if all the independent variables remain constant.

Model selection was based on lowest Akaike's Information Criterion (AIC) or Bayesian Information Criterion (BIC) and standardized Root Mean Square Errors (SRMSE) of prediction. Residuals diagnoses were performed to examine and validate a good fit of the model using sequence plots to ensure sufficiency of model and constant variation of errors, and residual normality plots to examine normal distribution of errors. Furthermore, plots of fitted versus reported dengue cases were also examined for good fit of the model.

### Model validation and forward forecast of dengue cases in 2011 and in 2012

Upon selection of a model that best described the data based on 2000–2010 dengue cases, we used the model to forecast cases for years 2011 and 2012. In the first 16 weeks of 2011, we used data in the last quarter of 2010 to forecast dengue incidence from January–April 2011. Subsequently, we input only weather data for January–December 2011 and prompt our model to forecast dengue cases from week 17 of 2011 to week 16 of 2012. Only weather data that were known at the time of issuing the 16 weeks forecast were used. Forecasted dengue cases in each period were then computed as autoregression for subsequent 16-week forecast. The forecast was repeated iteratively over time to generate the forecast for 2011–2012. Finally, we analyzed forecast precision by comparing forecasted cases against real-time clinical and laboratory-confirmed dengue cases (external data) reported by the MOH in each week. We also performed sensitivity tests on these data.

### Sensitivity tests based on ROC curve

An effective dengue forecast provides accurate information and minimizes false alarms so as to reduce unnecessary wastage of limited resources. We therefore further identified the optimal model using C-statistics or a Receiver Operating Characteristics (ROC) curve to evaluate and compare the sensitivity of the selected model in detecting true dengue outbreaks during both the model development and forecasting periods. The ROC curve analyzes the sensitivity or true positive rate of a model to predict outbreaks versus the false positive rate (1-specificity). The area of the ROC curve is the proportion of accurate prediction and this measures overall ability of a model to distinguish between a true outbreak and non-outbreak. We obtained annual outbreak or epidemic thresholds that were available for 2004–2011 from epidemiological reports published by the MOH Singapore. The local authorities computed warning level and epidemic threshold annually and dengue epidemic would be declared if total weekly cases exceed the epidemic threshold. We computed binary outcome of positive or negative outbreaks in each year based on given epidemic threshold values.

## Results

**Figure 1:**
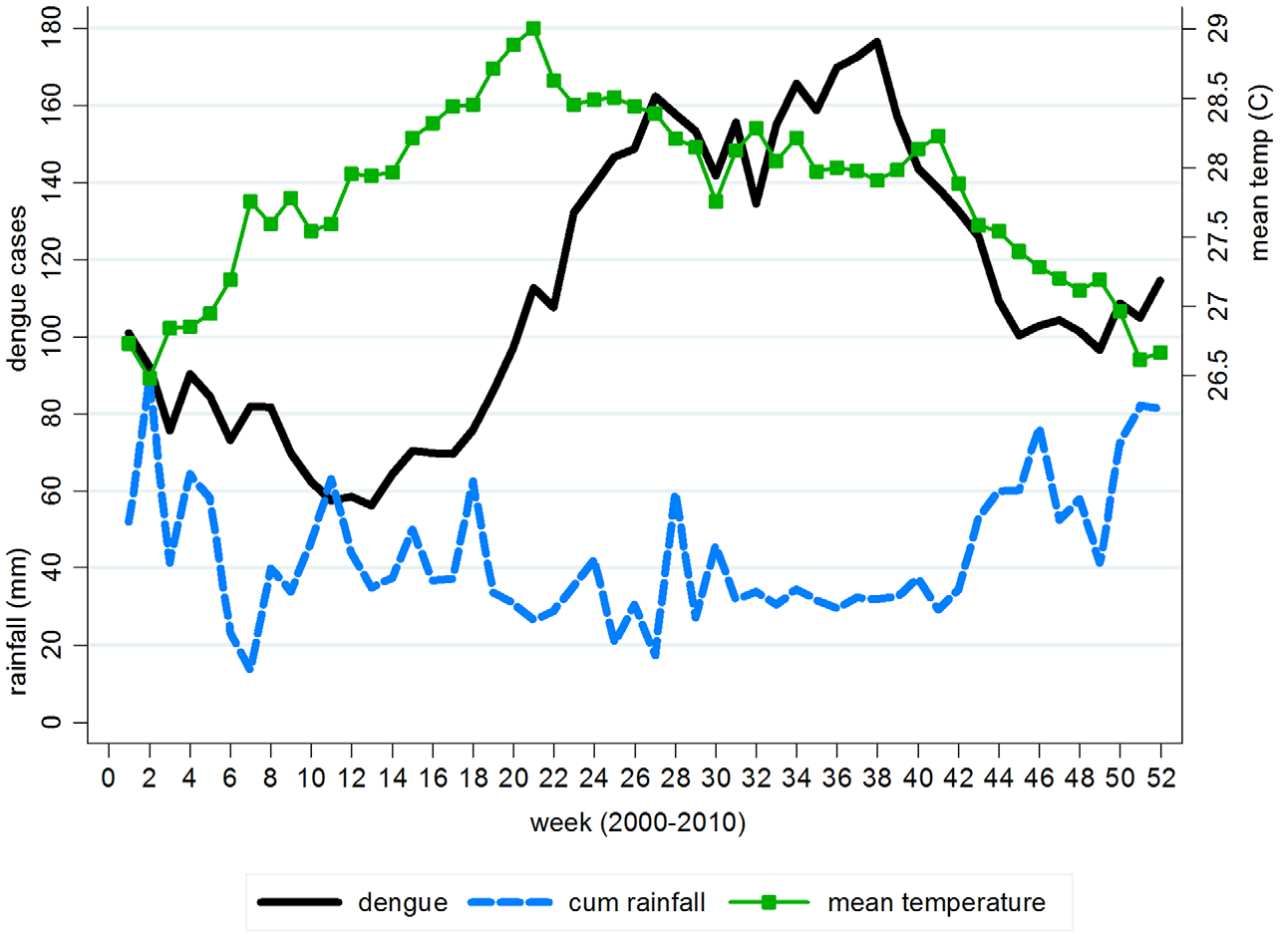
Average weekly distribution of dengue cases, mean temperature, and cumulative rainfall. Graphical presentation of lag relationship between weather predictors and dengue cases using weekly average over the period 2000–2010.

During the study period, the heaviest rainfall occurred in December (max = 394 mm, mean = 70 mm, std dev = 74 mm); whereas the highest temperature occurred in May (max = 30.3°C, mean = 28.7°C, std dev = 0.7°C). As shown in Figure 1, the average weekly mean temperature increased from week 1 and peaked at week 21 before declining gradually to the end of the year, whereas rainfall, which had less distinctive pattern generally demonstrated a wide ‘U’ pattern with the lowest amount of rainfall during weeks 19–42. Dengue incidence was generally higher during June–October period or between week 23 and 43, except in 2005 when Singapore experienced a dengue outbreak in early 2005 which was a spillover from the end of 2004. From 2000–2010, dengue outbreaks occurred in years 2004, 2005, and 2007.

Our findings showed that dengue cases with 6 weeks serial relationship best fitted the selected model. The cross correlation between temperature and dengue cases showed a symmetrical sine wave oscillating about the zero line at a time frame of about 24 weeks per cycle. The symmetrical pattern suggested a consistent and stable relationship between mean temperature and dengue incidence; indicating that mean temperature could be a strong predictor for dengue forecast. Simultaneously, cross correlation between weekly cumulative rainfall and dengue revealed asymmetrical oscillation at less consistent time cycles. Our findings showed that a model using weather time cycle of 20–24 weeks at lag term of 16 weeks performed with consistency during both training and forecast periods compared with models with other lag terms and time cycles. We selected the model that exhibited consistency in performance, high prediction precision, and lowest SRMSE in the forecast period. Standardized prediction errors (SRMSE) of the selected model were 0.3 and 0.32 of the standard deviation of reported dengue cases during the model development period (2000–2010) and forecast in 2011–2012, respectively. The SRMSE can be interpreted as the average error in the forecast of weekly dengue counts. Weather time cycles included in the selected model were 24 weeks for mean temperature and 20 weeks for rainfall.

According to our findings, the autoregressive term (k) in equation (1) v = 6; lag term (L) in equation (2) and (3) = 16; time cycle of mean temperature (n) in equation (2) = 24; and time cycle for rainfall (m) in equation (3) = 20.

**Figure 2:**
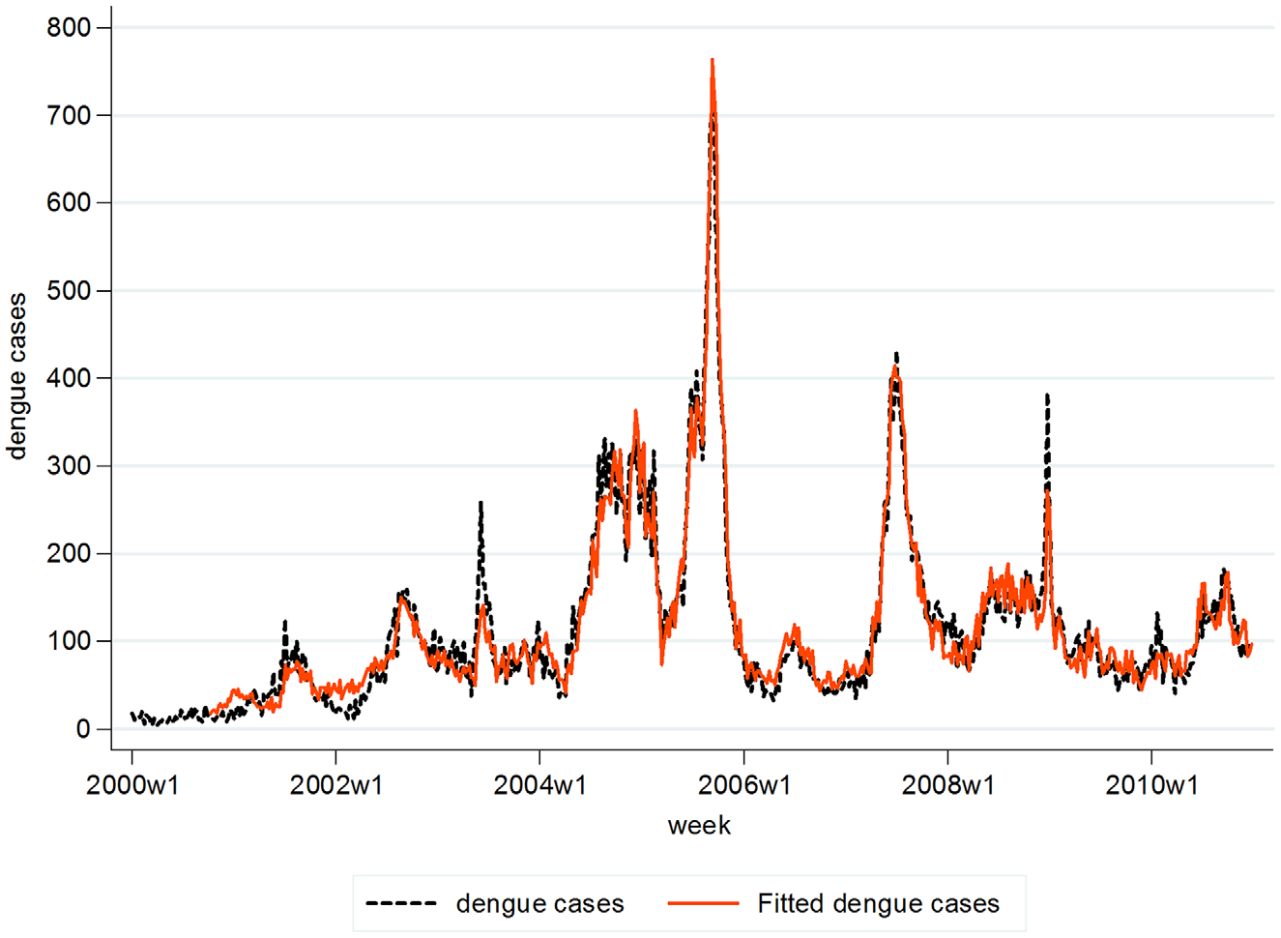
Fitted dengue cases versus reported dengue cases in 2000–2010. Model-based predicted or fitted dengue cases were plotted against actual reported dengue cases during the model training period.

The R^2^ (0.84) of our model suggests that mean temperature, rainfall, past dengue cases, season and trend explained 84% of the variance of weekly dengue distribution. The time series of fitted cases against actual reported cases as shown in Figure 2 exhibited a good fit of the model. The model was able to predict the peaks of the outbreaks that occurred in years 2004, 2005, and 2007. A scatter plot of fitted versus reported dengue cases illustrated that most of the fitted cases are scattered about the zero value with constant variance; suggesting no violation of model assumptions. Residual histograms exhibited a single modal and almost symmetrical pattern, the residual normal probability plot presented a reasonably straight line, and a residual sequence plot showed that distribution is consistent about the zero value and within upper and lower limits of +/−100. Thus, suggesting approximate normal distribution of residuals.

### Forecast of dengue cases in 2011 and 2012

**Figure 3:**
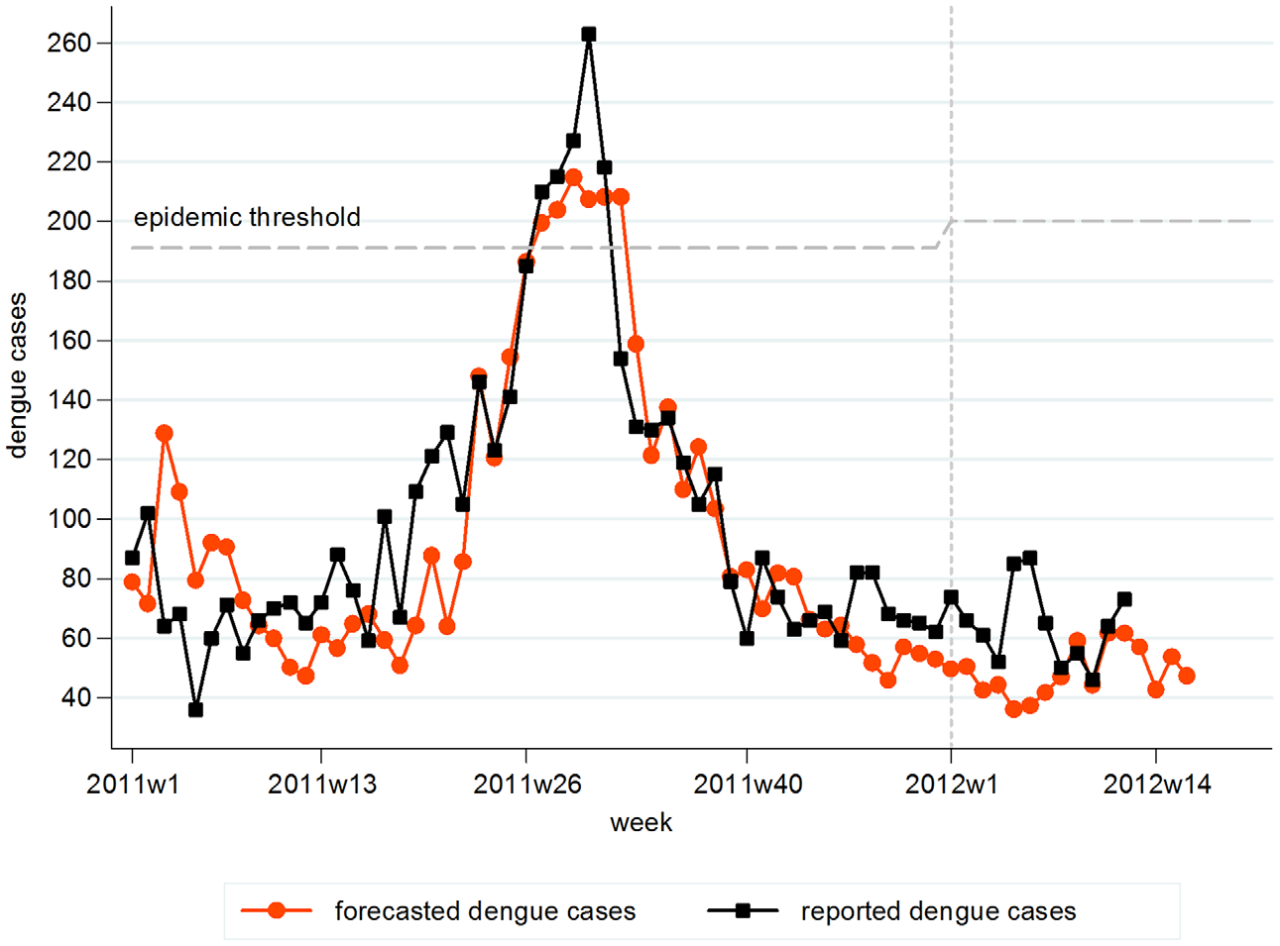
Forecasted dengue cases versus reported dengue cases in 2011–2012. Weekly forecasted dengue cases compared with reported cases during the validation period from 2011 week 1 to 2012 week 16. Epidemic threshold was 191 cases for 2011 and 200 cases for 2012.

During the forecast for 2011–2012, the optimal model forecasted cases versus actual clinical reported dengue cases gave an average error of 0.32 of the standard deviation of reported cases. As shown in Figure 3, the model forecasted cases with lower errors against actual reported cases in the 2^nd^ half of the year. In 2011, reported clinical cases exceeded the epidemic threshold for 5 consecutive weeks between weeks 27 and 31. Our model forecasted all the cases above the epidemic threshold with one false positive case at week 32. We have matched our forecast against external data or the real-time reported weekly cases from MOH up to week 12 of 2012; thus far, the model forecasted dengue incidence within the estimated range of errors.

**Figure 4:**
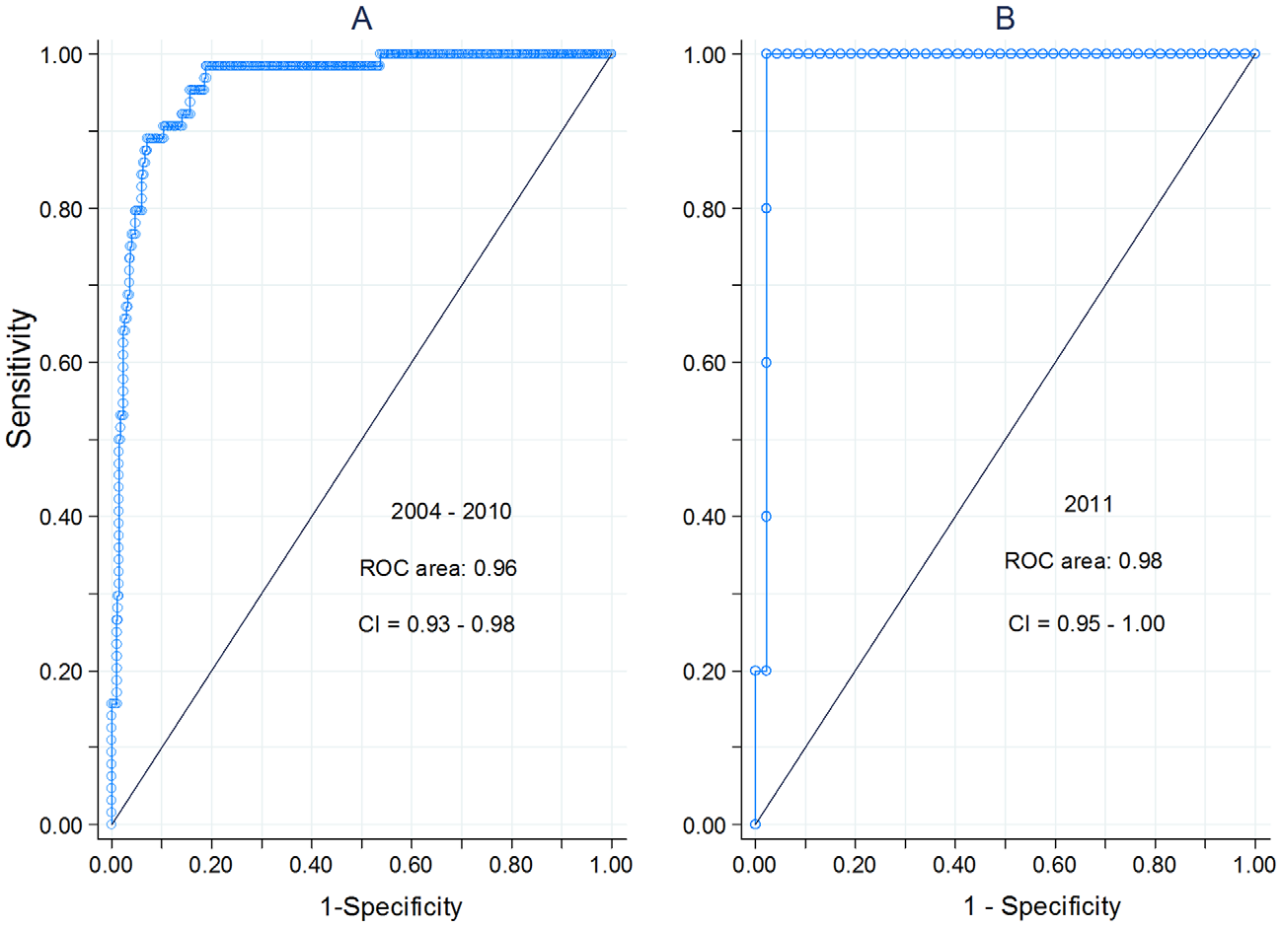
Analysis of sensitivity of model to detect reported dengue epidemics using ROC curves. ROC curves in graph A and B show sensitivity of model to detect true outbreak with corresponding probability of false alarm in year 2004–2010 and 2011, respectively.

ROC analysis suggested that our model performed with sensitivity ranging from 98–99% during outbreaks in 2004, 2005, and 2007. Estimated ROC areas for the period 2004–2010 indicated that the selected model performed at about 96% (CI = 93%–98%) sensitivity in distinguishing between outbreaks and non-outbreaks (Figure 4: Graph A), and in 2011 forecast with 98% (CI = 95%–100%) sensitivity in detecting a true outbreak (Figure 4: Graph B). ROC curves as shown in Figure 4 suggest a sensitivity for diagnosing true outbreaks between 90% and 98% during years 2004–2010 corresponding with a 10% to 20% risk of false alarm; whereas, in 2011 the forecasting model showed 100% sensitivity with less than 3% risk of false positive. Overall, the ROC suggested that the selected model performed consistently at above 90% during both model development and forecast periods.

## Discussion

Our model forecasted dengue cases up to 16 weeks ahead using retrospective weekly mean temperature and cumulative rainfall. It showed a consistent ability to separate weeks and years with epidemic and non-epidemic transmission in the training data, as well as outside the training time period in 2011. Based on lagged weather data and dengue counts the model predicted 5 out of the 5 epidemic weeks in 2011 correctly, using a 16 week lead time, thus, allowing sufficient time to prepare and potentially curb the epidemic. During the forecasting period in 2011, forecast precision based on prediction error (SRMSE) and sensitivity (ROC) tests suggested that the model forecast cases with high sensitivity for detecting outbreaks with a low risk of false alarms. The tests results during both training and forecast periods showed small discrepancy in SRMSE with absence of over fitting; thus demonstrating the stability of the model since the forecast in 2011 was performed without using actual reported cases as autoregression.

In recent years, the ability to predict local and regional weather in terms of accuracy and lead times has rapidly been improved due to advances in technology. This had allowed a better understanding of the interaction between climate and the temporal-spatial distribution of infectious diseases as well as stimulating research interest on epidemic prediction modeling [36]. We developed the weather-based dengue forecasting model based on scientific evidence that temperature and rainfall has significant influence on vectors and dengue viruses [21], [22], [23], [24], [35], [37], [38]. Dengue cases are influenced by complex interactions of ecology, environment, human, vectors, and virus factors. The lag time between weather and dengue cases could be partly accounted for by the impact of weather conditions on the biological development of the mosquito vector including long egg hatching periods and high possibility of *Aedes*' eggs to survive waterless for several months [21], [22], [23], [24], [35].

Several studies have documented relationship between weather variables and dengue cases in Singapore. In the late 90 s, a study that examined the links between dengue cases and *Aedes* mosquito population as well as weather conditions in Singapore shows that escalating temperature precedes rising dengue incidence by 8–20 weeks [16]. A recent study on the association between weather variables and dengue cases in Singapore using data from 2000–2007 has suggested that minimum and maximum temperature are strong weather predictors for the increase of dengue cases; whereas, rainfall and relative humidity have little correlation with dengue cases [39]. Using a different approach in study design, Hii *et al.* (2009) have quantified the effects of weekly mean temperature and cumulative rainfall on the risks of dengue cases across lag times up to 20 weeks [15], [27]. In their study they considered lag relationship between weather and dengue cases, impact of previous outbreaks on current number of cases, and influences of non-climatic factors. In addition, they applied smoothing functions to allow non-linear relationship between exposures (mean temperature and rainfall) and responses (risk of dengue cases) as well as adopted quasi-Poisson to permit over dispersion of data. Their findings show impacts of mean temperature and cumulative rainfall on risks of dengue cases vary according to each unit change in weather predictors in different lag windows (1–20 weeks). Overall, higher relative risks of dengue cases were identified at lag weeks 9–16. Evidence that weather is also a driver of dengue epidemics and trends of dengue has recently been confirmed by Descloux *et al.* (2012) in a study in New Caledonia [40]. It therefore seemed reasonable to assume that weather would be a precipitating factor in dengue epidemics in Singapore.

This study demonstrates that weather variables could be important factors for the development of a simple, precise, and low cost functional dengue early warning. A weather-based dengue early warning system could benefit local vector surveillance and control in several ways. First, an early warning system enhances efforts of dengue control to reduce the size of an outbreak which in turn decreases disease transmission, averts possible mortality, and lowers healthcare burden and operating costs incurred during an outbreak. Second, the use of publicly available weather variables removes the necessity for financial investment in weather-based predictive methods and further allows vector control units to focus their operations on high risk period; thus, maximizing limited vector control resources. Third, reports and study have suggested that local authorities require a maximum 3 months to curb a localized dengue outbreak [7], [27]. The forecast window of 16 weeks shown in this model offers ample time for local authorities to mitigate a potential outbreak effectively. Finally, high precision and sensitivity of a forecast minimizes the use of resources and prevents unnecessary vector control operations triggered by false alarms. Vector control can be resource and capital intensive; hence, high operating costs and unnecessary psychosocial stress in the population subsequent to false alarms could possibly hamper the decision to adopt a dengue early warning. Thresholds for true or false positive rates could vary according to scale of operational complexity and its consequences. We recommend an economic study on cost-effectiveness analysis to identify thresholds of true and false positive rates of forecast to serve as yardstick for decision making as well as to evaluate the long term benefits of an early warning against operating costs.

Nevertheless, a dengue forecasting model faces the challenge of long term sustainability of forecast precision since it assumes that a historic distribution pattern will be repeated in the future; while dengue epidemiology is influenced by a combination of factors which are dynamic and possibly evolving over time. Implementation of a new vector control policy could exert direct impact on the size of the vector population and dengue incidence rate in the locality. These changes are likely to influence the trend and epidemic cycle in the long run. Though changes of dengue distribution in the long term are inevitable due to the dynamics of disease transmission and changes of relevant policy, forecast errors can be minimized by making appropriate adjustment of the model through anticipating 1) changes in risk factors and 2) changes in related fields that will eventually influence the disease transmission. Therefore, current knowledge of factors influencing dengue distribution patterns can be used to re-calibrate the model in the future to maintain long term forecast precision.

A weather-based dengue forecast is often geographically fixed due to variability of local weather conditions. Likewise, the dynamics of dengue disease transmission in a community can be influenced by risk factors unique to that local context. Therefore, a locality based dengue forecast is usually applicable only to a specific study area. Nevertheless, the methodology of a weather-based dengue forecasting model could be extrapolated to other geographical areas. Partly due to an exponential growth of regional travels and trades, the Asia Pacific region has experienced an upsurge of dengue incidence in recent years. This suggests that a dengue endemic nation such as Singapore will no longer be able to curb or eliminate dengue without wider regional efforts. A regional dengue early warning system could signal risk of epidemic to all neighboring countries and help to prevent the regional chain effects of dengue outbreaks and so reduce the burden of dengue disease in neighboring countries. Therefore, a regional dengue forecast using weather anomaly such as El Nino index or sea surface temperature will inevitably complement and enhance the success of both national and regional dengue control.

In recent years, local authorities in Singapore heighten alert for the risk of increase in dengue cases as ambient temperature increases. Our study results demonstrate that a weather-based dengue forecasting model could provide more precise information on occurrence, timing, and size of dengue epidemics. A forecast that diagnoses outbreaks accurately and simultaneously gives about a four months window for implementing control measures could be invaluable in making control or even elimination of the cyclical dengue epidemic in Singapore a feasible possibility. We recommend a further study to analyze the possibility of incorporating a weather-based dengue early warning into the national dengue surveillance system. Further studies to improve long term sustainability of forecast precision will help to maintain the performance of a forecasting model. Moreover, a research to transform the forecasting model into a user-friendly or non-technical operational instrument comprehensible by users without specialist knowledge would encourage widespread adoption of such a dengue early warning system.
